# Ba_3_(PO_4_)_2_ Photocatalyst for Efficient Photocatalytic Application

**DOI:** 10.1002/gch2.202300257

**Published:** 2023-12-07

**Authors:** Yassine Naciri, Ayoub Ahdour, Elhassan Benhsina, Mahmoud Adel Hamza, Asmae Bouziani, Abdelghani Hsini, Bahcine Bakiz, Jose Antonio Navío, Mohamed Nawfal Ghazzal

**Affiliations:** ^1^ Institut de Chimie Physique UMR 8000 CNRS Université Paris‐Saclay Orsay 91405 France; ^2^ Laboratory of Materials and Environment Faculty of Sciences Ibn Zohr University B.P 8106 Agadir Morocco; ^3^ Materials Science Center Faculty of Sciences Mohammed V University in Rabat Rabat B.P:8007 Morocco; ^4^ Chemistry Department Faculty of Science Ain Shams University Abbasia Cairo 11566 Egypt; ^5^ Department of Chemistry School of Physics Chemistry and Earth Sciences The University of Adelaide Adelaide SA 5005 Australia; ^6^ Chemical Engineering Department Middle East Technical University Ankara 06800 Turkey; ^7^ National Higher School of Chemistry (NHSC) University Ibn Tofail BP. 133 Kenitra 14000 Morocco; ^8^ Laboratory of Advanced Materials and Process Engineering (LAMPE) Faculty of Science Ibn Tofail University BP 133 Kenitra 14000 Morocco; ^9^ Instituto de Ciencia de Materiales de Sevilla Centro Mixto Universidad de Sevilla‐CSIC Américo Vespucio 49 Sevilla 41092 Spain

**Keywords:** barium phosphate (Ba_3_(PO_4_)_2_), photocatalysis, photodegradation, rhodamine B, wastewater treatment

## Abstract

Barium phosphate (Ba_3_(PO_4_)_2_) is a class of material that has attracted significant attention thanks to its chemical stability and versatility. However, the use of Ba_3_(PO_4_)_2_ as a photocatalyst is scarcely reported, and its use as a photocatalyst has yet to be reported. Herein, Ba_3_(PO_4_)_2_ nanoflakes synthesis is optimized using sol‐gel and hydrothermal methods. The as‐prepared Ba_3_(PO_4_)_2_ powders are investigated using physicochemical characterizations, including XRD, SEM, EDX, FTIR, DRS, *J*–*t*, LSV, Mott‐Schottky, and EIS. In addition, DFT calculations are performed to investigate the band structure. The oxidation capability of the photocatalysts is investigated depending on the synthesis method using rhodamine B (RhB) as a pollutant model. Both Ba_3_(PO_4_)_2_ samples prepared by the sol‐gel and hydrothermal methods display high RhB photodegradation of 79% and 68%, respectively. The Ba_3_(PO_4_)_2_ obtained using the sol‐gel process exhibits much higher stability under light excitation after four regeneration cycles. The photocatalytic oxidation mechanism is proposed based on the active species trapping experiments where O_2_^•‒^ is the most reactive species. The finding shows the promising potential of Ba_3_(PO_4_)_2_ photocatalysts and opens the door for further investigation and application in various photocatalytic applications.

## Graphical abstract

Barium phosphate (Ba_3_(PO_4_)_2_) is a class of material that has attracted significant attention thanks to its chemical stability and versatility. In this work, the authors show that the synthesis method of Ba_3_(PO_4_)_2_ affects the photocatalytic activity. Thus, the sol‐gel process allows obtaining mutch higher photoactive process exhibits much higher photoactive Ba_3_(PO_4_)_2_ compared to the hydrothermal method.

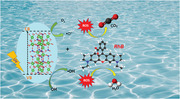

## 1 Introduction

Due to the rapid increase in the global population and industrial activities, the issue of air and water pollution has become a significant concern affecting human health.^[^ ^1^ ^]^ Among the various pollutants, organic textile dyes, including Rhodamine B (RhB) dye, are particularly common in wastewater from diverse industrial sources, posing a significant threat to aquatic life and human health.^[^ ^2^ ^]^ These dyes are often toxic, carcinogenic, and mutagenic, emphasizing the urgent need for effective methods to remove them from wastewater.

Textile waste can be treated through various traditional methods, including adsorption, filtration, reverse osmosis, chemical precipitation, solvent extraction, ion exchange, and, as well as bio‐/chemical degradation.^[^ ^3^, ^4^, ^5^, ^6^, ^7^, ^8^ ^]^ However, these methods are not highly efficient and often result in secondary pollution by transferring the organic pollutants to another phase.^[^ ^3^, ^4^ ^]^ Therefore, there is a critical need to explore novel reactive systems offering significantly higher efficiency than conventional purification processes. Recently, advanced oxidation processes (AOPs), including semiconductor‐assisted photocatalysis, demonstrated outstanding performance toward completely degrading and mineralizing recalcitrant organic pollutants.^[^ ^9^ ^]^ AOPs depend on producing highly reactive radicals, such as hydroxyl radicals (OH^•^), which play a crucial role in degrading organic pollutants through oxidative or reductive routes.^[^ ^10^, ^11^ ^]^ These processes offer a promising solution for efficiently removing organic dyes and other pollutants from wastewater.^[^ ^12^, ^13^ ^]^

Various semiconductors have been employed as photocatalysts, including metal oxides (e.g., TiO_2_, ZnO, WO_3_, etc.), metal sulfides (e.g., CdS, MoS_2_, etc.), and carbon‐based semiconductors (e.g., g‐C_3_N_4_, metal‐organic frameworks (MOFs), etc.).^[^ ^14^, ^15^, ^16^, ^17^, ^18^, ^19^, ^20^, ^21^, ^22^, ^23^ ^]^ Recently, phosphates‐based photocatalysts such as BiPO_4_ and Zn_3_(PO_4_)_2_ have attracted considerable attention due to their unique magnetic, electrical, optical, and photocatalytic properties.^[^ ^24^, ^25^, ^26^, ^27^, ^28^ ^]^ The phosphates‐based photocatalysts have shown promising ability toward degradation of organic pollutants.^[^ ^29^, ^30^, ^31^, ^32^ ^]^ The inductive effect of PO_4_^3−^ and the large dispersion of the conduction band to enhance electron–hole (e^−^/h^+^) pairs separation explains the good photoactivity in phosphate materials.^[^ ^28^ ^]^ Hence, using other new phosphate‐based photocatalysts would be promising to widen their use in photocatalytic redox reactions.

Barium phosphate, Ba_3_(PO_4_)_2_, is a promising phosphate‐based material, however, few papers have investigated its characteristics and applications. Zhang et al.^[^ ^33^ ^]^ studied the hydrothermal synthesis of strontium‐barium phosphate nanorods (Sr_3_(PO_4_)_2_, Ba_3_(PO_4_)_2_, and Ba_2.5_Sr_0.5_(PO_4_)_2_) and the removal of methyl blue dye via adsorption on these nano‐adsorbents. Then, Zhang et al.^[^ ^34^ ^]^ investigated the hydrothermal synthesis of magnetic Fe_3_O_4_@Ba_3_(PO_4_)_2_ nanoflakes loaded by Ag‐nanoparticles as well as their antimicrobial activity toward water disinfection via adsorption/degrading microorganisms such as E. *coli*. Recently, Obulapathi et al.^[^ ^35^ ^]^ investigated the structural and optical properties of Ba_3_(PO_4_)_2_ nanomaterials. The results showed that pH value in the wet chemical synthesis method has a critical effect on the final product's crystal structure, which may change from rhombohedral to orthorhombic, as well as its morphology. Additionally, the pH affects the optical bandgap energy to change from 4.89 eV at pH 7 (orthorhombic phase) to 3.60 eV at pH 13 (rhombohedral). Hence, the synthesis method of photocatalysts plays a crucial role in determining their effectiveness in photodegradation applications. The choice of synthesis method can influence factors such as particle size, crystal structure, surface area, and the presence of specific functional groups, all of which directly influence photocatalytic degradation efficiency. By carefully selecting and optimizing the synthesis method, researchers can enhance the photocatalytic activity of the materials, leading to improved degradation rates and higher levels of pollutant removal.

To the best of our knowledge, the photocatalytic activity of Ba_3_(PO_4_)_2_ was yet to be reported. Accordingly, there is no comparative study about the effect of the synthesis methods on the characteristics and photocatalytic performance of Ba_3_(PO_4_)_2_ nanoflakes. In this work, we have studied the impact of the synthesis method of Ba_3_(PO_4_)_2_ nanoflakes on the physicochemical properties and photocatalytic properties for oxidation reactions. Owing to their simplicity, efficiency, and low cost, sol‐gel and hydrothermal methods were employed for synthesizing Ba_3_(PO_4_)_2_ photocatalysts. The effect of the synthesis method on the Ba_3_(PO_4_)_2_ was investigated using various physicochemical characterizations, including XRD, SEM, EDX, FTIR, DRS, *J–t*, and EIS. The photocatalytic activities of the as‐synthesized Ba_3_(PO_4_)_2_ samples are assessed toward the photodegradation of RhB dye under UV irradiation. Finally, radical trapping experiments have been employed to elucidate reactive radical species and the mechanism of the photodegradation process.

## 2 Results and Discussion

### 2.1 Crystalline Properties

**Figure 1:**
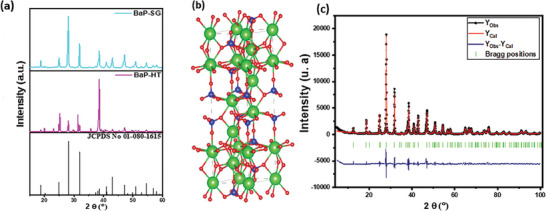
a) XRD patterns of the BaP‐SG and BaP‐HT, b) Schematic representation of Ba_3_(PO_4_)_2_ structure (green, blue, and red atoms represent Ba, P, and O, respectively). c) Refinement of the X‐ray diffraction and Bragg positions of Ba_3_(PO_4_)_2_.

The crystallinity and cell parameters of the synthesized BaP‐SG and BaP‐HT samples were examined. **Figure** **1**a displays the X‐ray diffraction (XRD) patterns of both samples. The XRD pattern of Ba_3_(PO_4_)_2_ obtained from the sol‐gel (BaP‐SG) and hydrothermal (BaP‐HT) methods exhibited multiple peaks located at 2*θ* values of 12.61°, 18.81°, 25.02°, 28.00°, 31.85°, 38.67°, 40.91°, 43.02°, 47.24°, 50.96°, 54.56°, 65.23°, and 66.85°. These peaks matched well with the characteristic planes of the trigonal phase of Ba_3_(PO_4_)_2_ (Figure 1b) with the *R* $\bar{3}$ *m* space group (JCPDS Card No. 01‐080‐1615). Using the conventional Scherrer equation, the average crystalline dimension (*D*) for the synthesized photocatalysts could be calculated from the full‐width at half maximum (FWHM) using Equation 1 ^[^ ^36^, ^37^ ^]^:

(1) D=(K·l)/(b·cosθ)

where *D* is the crystallite size, *K* is dimensionless constant (*k* = 0.9 for Gaussian profiles), *λ* is the X‐ray wavelength (1.54056 Å for CuKα radiation), *β* is the full‐width half maximum (FWHM) of the diffraction peak, and *θ* is the diffraction angle. The average crystalline sizes of the synthesized BaP‐SG and BaP‐HT photocatalysts are 27 and 64 nm, respectively. Moreover, the growth of BaP during the synthesis process shows a variable orientation plan as suggested by the intensity of the diffraction patterns. The relatively lower crystalline size of BaP‐SG compared to BaP‐HT suggests that BaP‐SG will exhibit higher surface area and better photocatalytic activity.^[^ ^38^ ^]^

The refinement of the structural parameters of the BaP‐SG (presumably the best photocatalyst) was done via Juna 2006 software, which permits the refinement of atomic coordinates, site occupancies, and atomic displacement parameters as well as profile parameters (instrument parameters, background parameters, lattice constants, and peak shape) (Figure 1c). Thus, the experimental difference and Bragg position could be calculated. The fitting of the diffractogram was done using the pseudo‐Voigt function, and the background was illustrated using linear interpolation between a set of manual points with refinable heights. The obtained parameters after refinement are summarised in **Table** **1**. The reliable accordance between the experimental and calculated pattern was assessed and a small difference between spectra and a well‐converged value of confident parameters was observed. The cell parameters were in agreement with the ones obtained in a previous study.^[^ ^39^ ^]^ The results show a good agreement between the experimental and the calculated pattern, highlighting the purity and homogeneity of the elaborated nanostructured powder.

**Table 1:** Crystallography data and refinement parameters of Ba_3_(PO_4_)_2_.

Crystallography data		
Chemical formula		Ba_3_(PO_4_)_2_
crystallin system		Trigonal
Molar weight		601.93 g mol^−1^
Space group		*R* $\bar{3}$ *m*
*V* (Å^3^)		573.117
Cell parameters	*a* (Ǻ)	5.6106 (6)
	*b* (Ǻ)	5.6106 (6)
	*c* (Ǻ)	21.023 (3)

Refinement		
Profile function		Pseudo‐voigt
Background		Linear intersection
Confident ratio	*R*_exp_	0.222
	*R*_p_	0.1707
	*wR*_p_	0.047
	*χ* ^2^	4.67

### 2.2 Morphological Properties

**Figure 2:**
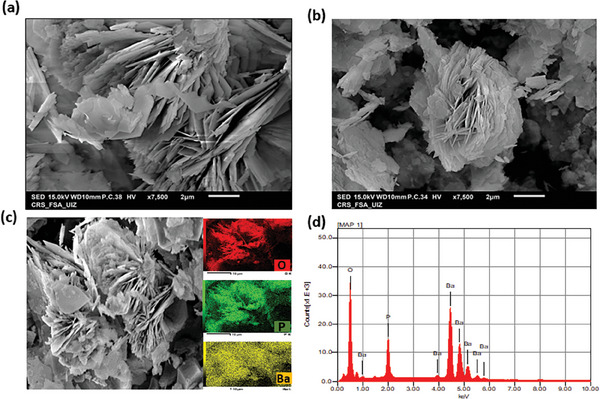
SEM micrographs of a) BaP‐SG, b) BaP‐HT, c) mapping micrographs, and (d) EDX spectra of BaP‐SG.

A scanning electron microscope (SEM) was used to identify the effect of the synthesis method on the morphology of the as‐synthesized Ba_3_(PO_4_)_2_ samples. **Figure** **2**a displays the images of the samples and reveals that BaP‐SG has uniform morphology and smaller particle size compared to the BaP‐HT sample (Figure 2b). Moreover, BaP‐SG exhibits irregular plate‐like particles, while BaP‐HT displays sharp flower‐like 3D formations, where platelet‐like particles are assembled to construct flower‐like microstructures (Figure 2b). Energy‐dispersive X‐ray spectroscopy (EDX) analysis was performed as mapping and determination of the elements of the as‐prepared materials as shown in Figure 2c,d. Peaks corresponding to Ba, P, and O elements were detected, indicating the presence of only Ba (23.81%), P (16.17%), and O (60.02%) in the synthesized BaP‐SG sample. No impurity peaks were observed, further confirming the purity of BaP‐SG. The Ba/P molar ratio is 1.47, which is in agreement with the theoretical molar ratio in Ba_3_(PO_4_)_2_.

### 2.3 Optical Properties and FT‐IR Results

**Figure 3:**
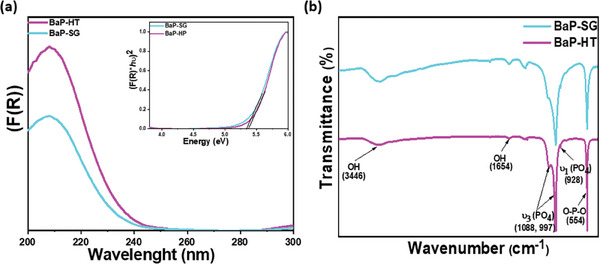
a) UV–vis spectrum of Ba_3_(PO_4_)_2_ prepared by SG and HT methods. Inset shows (α*hν*)^2^ versus *hν* plots of all samples, b) FTIR spectra BaP‐SG and BaP‐HT nanoparticles.

The optical properties of BaP‐SG and BaP‐HT photocatalysts were evaluated using UV–vis diffuse reflectance spectra (DRS). In both samples, the primary absorption was observed in the UV range (**Figure** **3**a). The BaP‐HT sample shows a lower wavelength onset of the absorption than the sol‐gel‐synthesized sample. The bandgap energy (*E*_g_) is determined using the Tauc's equation as for materials with a direct bandgap:

(2) $${\left(\alpha hu\right)}^{2}=K\left(hu-E_{g}\right)$$

where *K* is a constant and hν is the photon energy. An inset in Figure 3a presents the plot of (αhν)^2^ versus hν for Ba_3_(PO_4_)_2_ synthesized via sol‐gel and hydrothermal methods. The extrapolated straight line, fitted to the linear part of the curves, yields the value of Eg. The extracted values of *E*_g_ were found to be 5.34 eV for BaP‐SG and 5.37 eV for BaP‐HT. The slight variation in bandgaps among the materials synthesized by the two different methods translates to the change in particle size and their intrinsic physicochemical properties. Notably, the bandgap value is known to be highly dependent on particle size, resulting in a decrease in the bandgap with an increase in particle size.^[^ ^40^, ^41^ ^]^

The structural properties of Ba_3_(PO_4_)_2_ prepared by the sol‐gel and hydrothermal methods were investigated using Fourier‐transform infrared (FTIR) spectroscopy, as shown in Figure 3b. The spectrum reveals the band's appearance matching the different vibrations of the PO_4_^3−^ phosphate groups in the frequency scale 500–600 cm^−1^ and 920–1100 cm^−1^. In detail, the peaks centered at 1088, 997, and 928 cm^−1^ are assigned to the stretching vibrations of the P─O bond (*ν*_3_ and *ν*_1_) in PO_4_^3−^, while the vibrations (*ν*_4_) of O─P─O are at 554 cm.^−1[^ ^42^ ^]^ Additionally, two peaks observed at 1654 cm^−1^ and 3446 cm^−1^ are assigned to the deformation and elongation of O─H bonds of adsorbed water molecules.^[^ ^14^, ^43^, ^44^ ^]^

### 2.4 DFT Result

**Figure 4:**
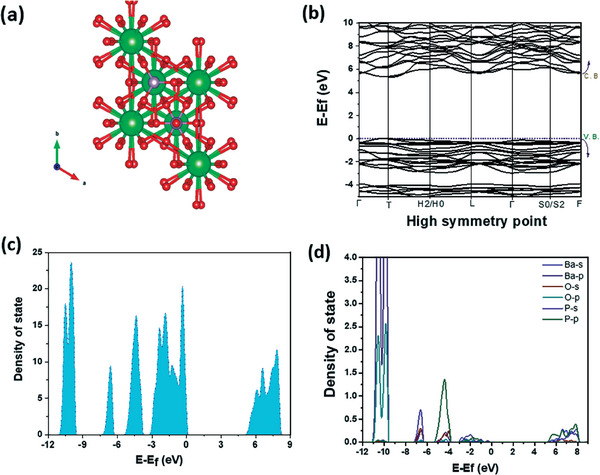
a) Optimized structure, b) band structure, c) total density of state and d) partial density of state of Ba_3_(PO_4_)_2_.

The cell parameters and atomic positions are firstly optimized, the estimated theoretical cell parameters and fractional atomic position are in good agreement with the experimental one (<2%). The computed unit cell volume (554.777 Å^3^) is determined to be smaller by ≈3.2% than the experimental volume (573.1717 Å^3^) (**Figure** **4**a).

The electronic structure of the phosphate Ba_3_(PO_4_)_3_ was investigated by the DFT approach, indicating the band structure (Figure 4b), total density state (Figure 4c) and projected density of state (Figure 4c) of the investigated phosphate. The valence band (VB) spreads from ≈ − 3.2 to 0 eV. The states within ≈3.2 eV below the valence band maximum (0 eV) are mainly contributed by 2p states of O atoms and a p state of phosphor atoms and barium atoms as shown in Figure 4d. The combination between 3p and 3s states of phosphorus, 2s and 2p orbitals of barium and 2s orbital of oxygen in the energy above ≈5.22 eV constitute the conductor band as depicted in Figure 4d. The computed bandgap is found to be ≈5.22 eV, which is in good agreement with the experimental gap of ≈5.3 eV. It may be noted that reasonable agreement of theoretical bandgap estimate with experimental bandgap may be a coincidence as DFT bandgapestimates are generally expected to be underestimated.

### 2.5 Photocatalytic Studies

#### 2.5.1 Photocatalytic Performance of the As‐Prepared Samples

**Figure 5:**
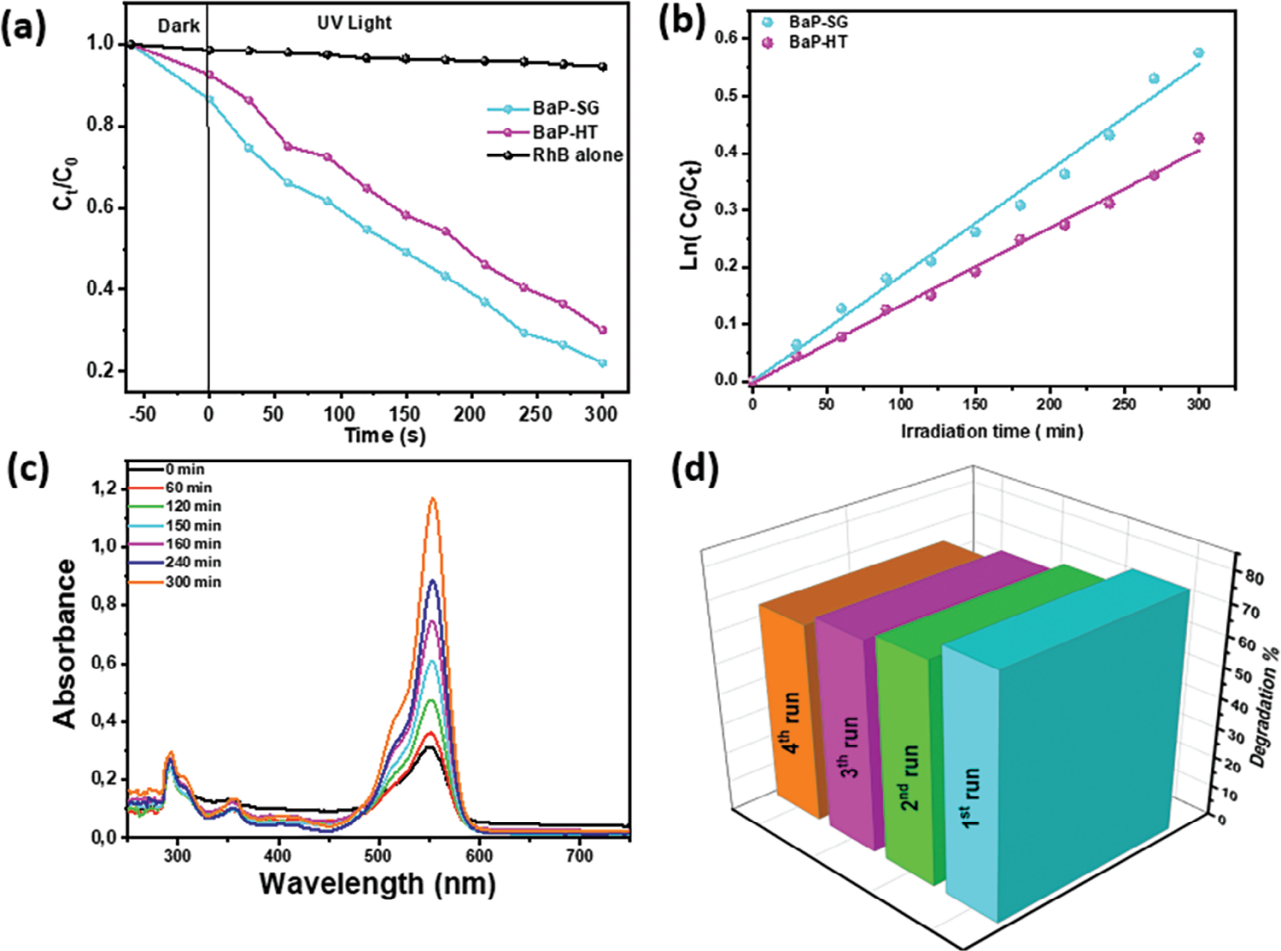
a) Variation in *C*_t_/*C*_0_ ratio as a function of time and b) pseudo‐first‐order kinetics of the photodegradation of Rhb dyne in the presence of BaP‐SG and BaP‐TH, c) UV–vis absorption with time irradiation of RhB in presence of BaP‐SG, d) cycling runs for the photocatalytic degradation of RhB. (Experimental conditions: [Cat] = 1 g L^−1^, [RhB]_0_ = 10 × 10^−6^ m, UV irradiation time = 300 min).

The study investigated the impact of the synthesis method of Ba_3_(PO_4_)_2_ on the photocatalytic activity of nanopowders for the degradation of RhB dye under UV irradiation. The time‐dependent photoactive performance of BaP‐SG and BaP‐HT photocatalysts, represented as photodegradation (%) of rhodamine B, is illustrated in **Figure** **5**a. RhB is known to be stable toward photolysis under UV‐irradiation (without photocatalyst), just 9% of RhB was removed after 300 min (Figure 5a). This is in good accordance with earlier reported works on RhB photodegradation.^[^ ^3^, ^23^ ^]^ For the same irradiation time, it was observed that the sol‐gel method yielded better activity compared to the hydrothermal method. The RhB degradation rate was 79% for BaP‐SG and 68% for BaP‐HT. Various researchers have reported that the sol‐gel method leads to more active material compared to the hydrothermal method, supporting the notion that the sol‐gel method is the preferred approach for synthesizing effective catalysts.^[^ ^38^, ^45^, ^46^, ^47^, ^48^ ^]^ We conducted a comparative analysis of our synthesized compound using both sol‐gel and hydrothermal methods with other phosphate‐based materials for the degradation of organic pollutants, as detailed in **Table** **2**. Notably, BaP exhibited a higher catalytic efficiency within a relatively shorter timeframe compared with other phosphate based photocatalysts strontium phosphate. For instance, stronium phosphate achieved a degradation rate of ≈ 68% for MO, 98% for RhB, and 90% for MB within a duration exceeding 4260  min (71 h)^[^ ^49^ ^]^ as shown in Table 2.

**Table 2:** Comparison of the photocatalytic activity of Ba_3_(PO_4_)_2_ with other phosphate‐based materials toward the photodegradation of organic pollutants expressed in removal efficiency (%) and the operation conditions were mentioned.

Phosphate based photocatalyst	Pollutant	Catalyst dose	Light irradiation	Removal efficiency [%]	Refs.
BiPO_4_	Sulfadiazine (10 mg L^−1^)	1 g L^−1^: 100 mg in 100 mL	UV light t = 240 min	≈62%	[29]
Zn_3_(PO_4_)_2_				≈42%	
Zn_3_(PO_4_)_2_/BiPO_4_				≈97%	
Zn_3_(PO_4_)_2_	Methylene blue (MB, 5 mg L^−1^)	Thin film with 100 mL dye solution	UV or visible light t = 240 min	42% (UV) 26% (visible)	[53]
Zn_3_(PO_4_)_2_@CNT				85% (UV) 53% (visible)	
CePO_4_	Rhodamine B (5 mg L^−1^)	1 g L^−1^: 100 mg in 100 mL	UV light t = 480 min	≈84%	[54]
Ca_3_(PO_4_)_2_	Rhodamine B (5 mg L^−1^)	1 g L^−1^: 100 mg in 100 mL	UV light t = 240 min	39%	[36]
Sr‐Ca_3_(PO_4_)_2_				76%	
CePO_4_	RhB (10 × 10^−6^ m, ≈5 mg L^−1^) MO (10 × 10^−6^ m, ≈3.3 mg L^−1^) MB (10 × 10^−6^ m, 3.2 mg L^−1^)	0.2 g L^−1^: 0.01 mg in 50 mL	UV light t = 4120 min	98.5% (RhB) 68.8% (MO) 90.4% (MB)	[49]
Ba_3_(PO_4_)_2_ BaP‐SG BaP‐HT	Rhodamine B (10 × 10^−6^ m, ≈5 mg L^−1^)	1 g L^−1^: 100 mg in 100 mL	UV light t = 300 min	79% (SG) 68% (HT)	This work

Langmuir–Hinshelwood kinetic model is frequently employed to explain the photocatalytic degradation process of RhB by catalysts.^[^ ^50^ ^]^ The first‐order reaction kinetic function can be expressed as ln(*C*_0_/*C*_t_) = *k*_app_ × *t*, where *C*_0_ is the initial RhB concentration, *C*_t_ is the concentration of RhB at the irradiation time, and *k*_app_ is the apparent constant rate of RhB degradation. Figure 5b shows the degradation of RhB over irradiation time and exhibits a well‐linear relationship, indicating pseudo‐first‐order kinetics. The *k*_app_ was estimated as 1.86 × 10^−3^ min^−1^ (*R* ^2^ > 0.98) and 1.36 × 10^−3^ min^−1^ (*R* ^2^ > 0.98) for BaP‐SG and BaP‐HT, respectively. Figure 5c represents the evolution of the RhB spectra during 5 h of irradiation in the presence of BaP‐SG photocatalyst. As illustrated, with prolonged irradiation time, the intensities of characteristic absorption peaks of RhB at 554 nm decrease gradually, implying the decrease of RhB concentration. The enhanced photocatalytic activity of BaP‐SG could be related to its larger surface area expected from the crystalline size. Accordingly, it is expected that the generation of reactive species would promote the decomposition of RhB molecules.^[^ ^51^, ^52^ ^]^ These results demonstrate that the photocatalytic activity of Ba_3_(PO_4_)_2_ considerably depends on the preparation methods.

The photostability and reusability of the BaP‐SG photocatalyst were evaluated via four sequential runs of the photocatalytic experiments. Figure 5d shows that the as‐synthesised BaP‐SG maintained its performance after four photocatalytic cycles (each for 300 min); only a slight decrease in the efficiency of the sample was noted. These findings point to the high stability and long durability of the as‐synthesized Ba_3_(PO_4_)_2_ photocatalyst and promote their potential applications.

#### 2.5.2 The Influence of pH on Photocatalytic Processes

**Figure 6:**
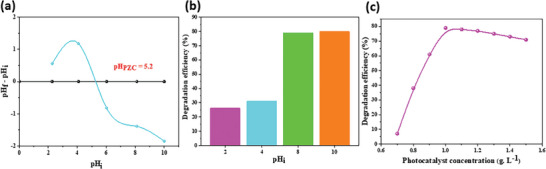
a) pH_PZC_ of the BaP‐SG compound, b) investigation of the impact of solution pH on RhB degradation using the BaP‐SG photocatalyst, c) investigation of the effect of BaP‐SG dosage on the photodegradation of RhB. (Experimental conditions: [Cat] = 0.7–1.5 g L^−1^, [RhB]_0_ = 10 × 10^−6^ m, pH = 2–10, UV irradiation time = 300 min).

The concept of the point of zero‐point charge (pH_PZC_) pertains to the equilibrium of charge species present on the catalyst's surface, leading to a state where the net electric charge density becomes zero. Put simply, it relates to the amphoteric characteristics of the catalyst, wherein acidic functional groups can balance out basic groups.^[^ ^55^, ^56^ ^]^ The pH_PZC_ of the effective BaP‐SG catalyst was practically determined using a facile pH drift method.^[^ ^23^, ^57^, ^58^ ^]^ As depicted in **Figure** **6**a, the ΔpH reached equilibrium at zero when pHi = pH_PZC_ = 5.2. Beneath this threshold (pH < pH_PZC_), the BaP‐SG surface bore a positive charge, whereas beyond it (pH > pH_PZC_), the catalyst's surface carried a negative charge. The exceptional photocatalytic efficiencies observed in the case of the cationic dye RhB can be attributed to the electrostatic attraction between the dye and the negatively charged BaP‐SG catalyst under the natural pH conditions (pH = 7). The effect of solution pH on photocatalysis for RhB removal was thoroughly investigated. Figure 6b illustrates the degradation rate of RhB under four different pH conditions (2, 4, 8, and 10) during 300 min of illumination. As observed, the photocatalytic activities of the dye were most pronounced in basic solutions (pH = 8 and 10), achieving a peak efficiency of 80% at pH = 10. Conversely, the efficiency significantly dropped to less than 26% in more acidic environments. In conclusion, when the pH falls below pH_PZC_ = 5.2, it is anticipated that the catalyst's surface assumes a positively charged state, thereby intensifying electrostatic repulsion between the catalyst and the positively RhB molecules (COOH p*k*_a_ = 3.7) .^[^ ^3^, ^4^, ^23^, ^57^ ^]^ The extent of RhB degradation under natural pH conditions (pH = 6) closely resembles that in high pH environments. Consequently, we have opted to maintain the natural conditions due to their cost‐effectiveness, as they yield comparable results.

#### 2.5.3 The Impact of Photocatalyst Mass on Photocatalytic Reactions

In order to economically remove dye from wastewater, it becomes imperative to identify the optimal catalyst dosage for effective degradation. Our investigation explored the influence of photocatalyst concentration on the degradation of RhB, varying from 0.7 to 1.5 g L^−1^. The correlation between RhB photodegradation efficiency and photocatalyst concentration is depicted in Figure 6c. The results show that the photodegradation efficiency of RhB exhibits a rapid ascent from 7% to 79% as the concentration of BaP‐SG increases from 0.7 to 1 g L^−1^. Beyond this concentration (1 g L^−1^), a slight decline in the photodegradation efficiency of RhB is observed. This trend can be attributed to multiple factors. The escalation in BaP‐SG concentration augments the number of BaP‐SG particles, leading to an increase in photon absorption and subsequent degradation of RhB molecules. However, exceeding the 1 g L^−1^ threshold may introduce light scattering and screening effects.^[^ ^3^, ^4^, ^23^ ^]^ Excessive opaqueness within the suspension hinders the catalyst located farthest from the light source from being effectively illuminated. Consequently, the scattering and screening effects diminish the specific activity of the catalyst.^[^ ^59^ ^]^ Moreover, at high catalyst concentrations, particle aggregation may further impede catalytic activity. Hence, our study concludes that the optimal catalyst dosage for RhB degradation is 1 g L^−1^, as it strikes the balance between enhanced degradation efficiency and the onset of light scattering and screening effects.

### 2.6 Photoelectrochemical and Mechanism of Photocatalysis

**Figure 7:**
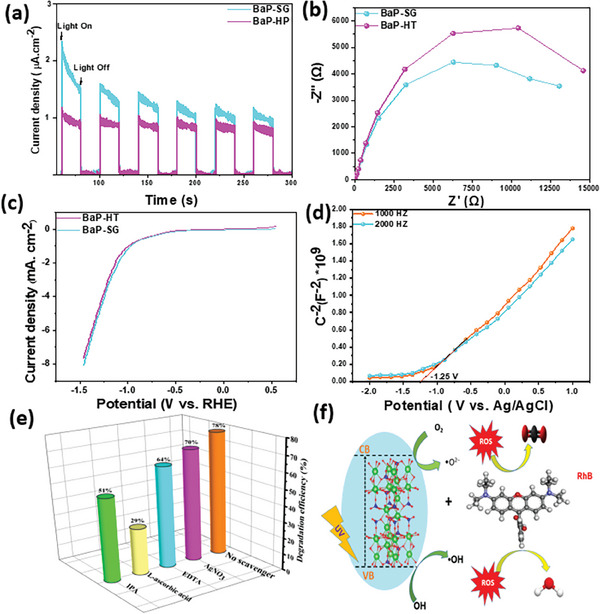
a) Photocurrent responses, b) EIS and c) LSV results of BaP‐SG and BaP‐HT under UV light irradiation, d) Mott‐Schottky results of BaP‐SG and BaP‐HT, e) effect of scavengers on the photocatalytic degradation of RhB in the presence of BaP‐SG (Experimental conditions: [Cat] = 1 g L^−1^, [RhB]_0_ = 10 × 10^−6^ m, UV irradiation time = 300 min), and f) Schematic diagram representing the proposed degradation mechanism of BaP‐SG.

The generation and separation of photogenerated charge carriers can be indirectly understood through the analysis of transient photocurrent generation.^[^ ^43^, ^60^ ^]^ **Figure** **7**a presents the transient photocurrent density–time (*J*–*t*) curves for BaP‐SG and BaP‐HT. Both samples exhibit rapid and consistent photocurrent behavior. However, the BaP‐SG electrode demonstrates a higher photocurrent than BaP‐HT, suggesting reduced electron‐hole recombination and enhanced photocatalytic activity under UV‐light exposure.

Electrochemical impedance spectroscopy (EIS) is commonly employed to investigate charge transfer processes by examining the resistance at the electrode/electrolyte interface. EIS spectra typically display semicircles, with the radius of these semicircles reflecting the resistance of the interface layer on the electrode surface. A smaller semicircle radius indicates higher charge transfer efficiency.^[^ ^16^, ^61^ ^]^ Figure 7b represents the EIS Nyquist plots for BaP‐SG and BaP‐HT, with the semicircle associated with BaP‐SG being smaller than that of BaP‐HT, indicating a lower charge transfer resistance. The results suggested that BaP‐SG possess fast interfacial electron transfer and exhibits a lower recombination rate, aligning with its superior photocatalytic activity. The EIS findings are consistent with the *J–t* results.

Besides *J–t* and EIS, the linear sweep voltammetry (LSV) method is also efficient in examining the charge separation efficiency of a photocatalyst. Figure 7c displays the LSV curves of BaP‐SG and BaP‐HT in 0.5 M Na_2_SO_4_ electrolyte. As could be seen from the figure, the BaP‐SG exhibited an increased photocurrent density compared to the BaP‐HT, which was consistent with the *J–t* and EIS results. The higher intensity of the current peak of BaP‐SG suggests a higher amount of photogenerated carriers, thus the best photocatalytic performance.

To determine the flat band potentials (*E*_fb_) of the as‐prepared photocatalyst, Mott‐Schottky (M‐S) measurements were performed on BaP‐SG at both 1000 Hz and 2000 Hz. The resulting plot and the derived flat band potential value are shown in Figure 7d. The positive slope of the M‐S curve for BaP‐SG indicates that it exhibits properties of an n‐type semiconductor. The *E*_fb_ of the BaP‐SG photocatalyst is determined to be −1.25 V relative to the Ag/AgCl electrode potential, which is equivalent to −1.11 V versus the normal hydrogen electrode (NHE). For n‐type semiconductors, the flat band potential is typically located approximately 0.3 V below the conduction band minimum.^[^ ^62^, ^63^ ^]^ Therefore, the conduction band potential of BaP‐SG is calculated to be −1.41 V. According to the UV–vis diffuse reflectance spectra (Figure 3a), the bandgap (*E*_g_) of BaP‐SG is determined to be 5.34 eV. Consequently, the valence band (VB) of BaP‐SG is calculated to be 3.82 eV using the equation VB = *E*_g_ + CB. The higher conduction band position indicates a stronger reductive power, which may be effectively involved in the photocatalytic reaction. The standard redox potential of O_2_/O_2_^•‒^ (−0.28 V vs NHE) is more positive than the ECB of BaP‐SG (−1.41 V), suggesting that the photogenerated electron could theoretically react with the adsorbed O_2_ to form O_2_^•‒^.^[^ ^64^ ^]^

Studying the photocatalytic degradation mechanism of RhB molecules using BaP‐SG photocatalyst under UV irradiation is of pivotal significance. Different scavengers were used to conduct the trapping experiments, such as isopropanol (IPA), EDTA‐2Na (EDTA), L‐ascorbic acid, and silver nitrate (AgNO_3_) were used to quench hydroxyl radicals (OH^•^), hole (h^+^), superoxide anion radicals (O_2_^•−^), and electrons (e^−^).^[^ ^3^, ^4^ ^]^ As shown in Figure 7e, the degradation efficiency drops from 78 to 29% and 51% after adding L‐ascorbic acid and IPA, respectively, indicating that both OH^•^ and O_2_^•‒^ play a crucial role in the photodegradation process. Nevertheless, the removal of RhB still exhibits a notable efficiency of 64% and 70% in the presence of EDTA and AgNO_3_, respectively. This suggests that the involvement of *h* ^+^ and e^−^ charge carriers in the degradation process is not direct. Thus, OH^•^ and O_2_^•−^ are the main active species in the photodegradation mechanism. Based on the above result, the predominant photocatalytic degradation pathway is given in Figure 7f following equations:^[^ ^3^, ^15^, ^65^ ^]^

(3) $${\mathrm{Ba}}_{3}{\left({\mathrm{PO}}_{4}\right)}_{2}+h^{+}\to {\mathrm{Ba}}_{3}{\left({\mathrm{PO}}_{4}\right)}_{2}\left(e^{-}+h^{+}\right)$$

(4) $$O_{2}+e^{-}\to O_{2}^{•-}$$

(5) $$O_{2}^{•-}+2H^{+}+e^{-}\to H_{2}O_{2}$$

(6) $$H_{2}O_{2}+e^{-}\to {\mathrm{OH}}^{•}+{\mathrm{OH}}^{-}$$

(7) $$H_{2}O+h^{+}\to H^{+}+{\mathrm{OH}}^{•}$$

(8) $${\mathrm{OH}}^{-}+h^{+}\to {\mathrm{OH}}^{•}$$

(9) $$O_{2}^{•-}+\mathrm{RhB}\to {\mathrm{RhB}}^{-}\to \mathrm{Degradation}\;\mathrm{products}$$

(10) $${\mathrm{OH}}^{•}+\mathrm{RhB}\to {\mathrm{RhB}}^{-}\to \mathrm{Degradation}\;\mathrm{products}$$

To summarize, the photocatalytic activity of BaP‐SG synthesized by the sol‐gel method increased compared to the BaP‐HT obtained from hydrothermal due to the smaller crystallite size of about 27 nm. The decrease in the crystallite size will increase the surface area. It is known that the photocatalytic redox reaction mainly takes place on the photocatalysts' surface, so the surface properties significantly influence the efficiency of the photocatalyst.^[^ ^48^, ^66^ ^]^ Conversely, morphology also represents a potential factor influencing the final degradation efficiency, which was reported earlier.^[^ ^38^, ^52^, ^67^, ^68^ ^]^ Moreover, the EIS, LSV and TPC were in good accordance with the photocatalytic results of RhB photodegradation. Finally, it is believed that this work will open the door for further investigation of various Ba_3_(PO_4_)_2_ photocatalysts for enhancing the photocatalytic performance via various techniques.

## 3 Conclusion

The hydrothermal and sol‐gel conditions influence the growths of the Ba_3_(PO_4_)_2_ photocatalyst, thus, significantly tuning their physicochemical properties. The crystal growth of the material had the utmost effect on the surface properties and the phase composition and, consequently, a critical influence on the photocatalytic activity. The sol‐gel method gave a photocatalyst with a higher degradation rate. We proposed that its high photocatalytic activity can be ascribed to the high photogenerated e‐h pair separation efficiency. Moreover, the scavenger experiments indicated that OH^•^ and O_2_^•‒^ play a crucial role in the photodegradation process. The results of reusability experiments demonstrated that BaP‐SG had good stability after four cycles of photodegradation. The results of this study show that Ba_3_(PO_4_)_2_ is an effective and environmentally friendly photocatalyst that can open possibilities to engineer a photocatalyst for feasible applications in wastewater purification.

## 4 Experimental Section

### Sol‐Gel Synthesis of Ba_3_(PO_4_)_2_ (BaP‐SG)

Nanostructured barium phosphate powder was synthesized using a typical sol‐gel process. The starting products were barium nitrate (Ba(NO_3_)_2_) (Sigma‐Aldrich, 98%) and ammonium dihydrogen phosphate (NH_4_H_2_PO_4_) (Sigma‐Aldrich, 98%), citric acid was used as a complex agent, and ammonia was used to justify the solution pH; all the used compounds are analytic purity. Each proper amount of reactant was dissolved separately in deionized water. After stirring, citric acid was added to the Ba(NO_3_)_2_ solution to form Ba^2+^ chelate. The phosphate solution was added dropwise to the barium solution. The resulting nanostructured barium phosphate powder was obtained through filtration. Subsequently, the powder was treated at 200 °C to remove any residual moisture. Equation 11 represents the general chemical reaction as follows:

(11) $$\begin{array}{c} \mathrm{Ba}{\left({\mathrm{NO}}_{3}\right)}_{2}+2{\mathrm{NH}}_{4}H_{2}{\mathrm{PO}}_{4}\to {\mathrm{Ba}}_{3}{\left({\mathrm{PO}}_{4}\right)}_{2}+2{\mathrm{NH}}_{4}{\mathrm{NO}}_{3}+4{\mathrm{HNO}}_{3} \end{array}$$

### Hydrothermal Synthesis of Ba_3_(PO_4_)_2_ (BaP‐HT)

For the preparation of Ba_3_(PO_4_)_2_ powder using the hydrothermal method, the same precursors, Ba(NO_3_)_2_ and NH_4_H_2_PO_4_, were employed in the sol‐gel route. The hydrothermal reaction was carried out in a 23 mL Teflon‐lined autoclave filled to 50% with distilled water, and the reaction was conducted under autogenous pressure at 110 °C for 24 h. Following the hydrothermal reaction, the mixture was gradually cooled to room temperature before their extraction, filtration and washing.

### Physicochemical Characterizations

Several techniques were employed to characterize the synthesized samples' physicochemical properties. X‐ray diffraction (XRD) patterns were obtained using an Empyrean Panalytical diffractometer with Cu Kα radiation at 45 kV/35 mA. The powders' local composition, morphology, and grain size were examined using scanning electron microscopy (SEM) (Supra 40 VP Column Gemini Zeiss) operating at 40 KeV, coupled with an EDS manifolder for quantitative elemental composition analysis. Fourier‐transform infrared (FTIR) spectroscopy was conducted using the SHIMADZU IRAffinity‐1S instrument to identify the functional groups present in the molecules. The samples were analyzed in the frequency range of 400–4000 cm^−1^ with a resolution of 4 cm^−1^. Light absorption properties of the samples were studied by UV–vis spectroscopy. The diffuse reflectance UV–vis spectra (UV–vis DRS) were recorded on a Varian spectrometer model Cary 100 equipped with an integrating sphere and using BaSO_4_ as a reference.

### Computational Details

The DFT calculations in this project were done using Quantum espresso code Ref. [69]. The exchange and correlation functional of Kohn‐Sham equations was achieved through the Generalized‐Gradient‐Approximation (GGA) using Perdew‐Burke‐Ernzerhof (PBE) parameterization with projector augmented waves (PAW).^[^ ^70^, ^71^ ^]^ k‐points grid of 7 7 7 centred according to the method of Monkhorst Pack^[^ ^71^ ^]^ was used to sample the Brillouin zone, the cutoff energy was sat to be 400 eV. The structural parameters were optimised with the proviso residual forces less than 0.08 eV Å^−1^. (VESTA) and XcrysDen software were used for the visualization of electronic and structural analysis. The orbitals of elements constituting the studied systems are Ba (5p, 6s), P (3p, 3s), and O (2p, 2s).

### Photoelectrochemical Measurements

The photoelectrochemical performance of the synthesized Ba_3_(PO_4_)_2_ samples was evaluated using a CHI 600E electrochemical workstation. Transient photoelectrochemical measurements were conducted in a three‐electrode cell containing a 0.5 m Na_2_SO_4_ solution, and the cell was illuminated with simulated sunlight (AM 1.5G, 100 mW cm^−2^). The working electrode consisted of the as‐synthesized Ba_3_(PO_4_)_2_ coated on FTO glass slides (2 cm × 1 cm).

The linear sweep voltammograms (LVSs) were measured from 0 to −2 versus Ag/AgCl with a scan rate of 5 mV s^−1^. Electrochemical impedance spectroscopy (EIS) tests were conducted in the same configuration at *n* = −0.6 V versus Ag/AgCl from 10^5^–10^−1^ Hz. Transient photocurrent measurements were carried out at 0.7 V versus Ag/AgCl. All potentials were calibrated with the RHE using the equation *E*_VS.RHE_ = *E*_vs.Ag/AgCl_ + *E*_vs.Ag/AgCl_+0.059 pH.

### Photocatalytic Test

The photocatalytic activities of the BaP‐SG and BaP‐HT samples were evaluated for the degradation of aqueous rhodamine B (RhB) solutions. Commercial Puritek 35 W lamps were employed as the UV light source. In a typical batch reactor, 100 mg of photocatalyst powder was dispersed in 100 mL of a 10 × 10^−6^ m RhB solution.

Before illumination, the suspensions were continuously stirred for 1 h to establish solid‐liquid adsorption‐desorption equilibrium. The temperature of the suspension was kept at 25 ± 1 °C. Subsequently, the UV light was turned on to start the photocatalysis process. At specific time intervals of 1 h, approximately 2.5 mL of the solution was collected and then filtered through a Millipore filter with a pore size of 0.22 µm to remove the catalyst particulates for further analysis.

A UV–Vis spectrophotometer (UV‐2300) was used to evaluate the concentration of RhB by following the absorbance at 554 nm. The RhB photodegradation percentages (*D*%) were calculated based on the change in concentrations over the irradiation time using Equation 12:^[^ ^15^, ^36^ ^]^

(12) $$D\;\left(\%\right)=\frac{C_{0}-C}{C_{0}}\;*100$$

where *C*_o_ and *C* are the concentrations of the solutions before and after UV irradiation.

### Determination of Point of Zero Charge (pH_PZC_)

The pH_PZC_ of the effective BaP‐SG catalyst was practically determined using a facile pH drift method.^[^ ^23^, ^57^, ^58^ ^]^ Typically, a series of five beakers were prepared, each with a distinct pH value (2, 4, 6, 8, and 10), all within a 0.5 m NaCl solution. After 48 h of continuous stirring, the pH_f_ was measured, and the results were presented as a plot illustrating the difference, denoted as ΔpH = pH_f_–pH_i_.

## Conflict of Interest

The authors declare no conflict of interest.

## Author Contributions

Y.N., A.A., and E.B. performed conceptualization and methodology. M.N.G., A.H., B.B., and J.A.N. administered the project. Y.N., A.B., and M.A.H. wrote and prepared the original draft. Y.N., M.A.H., and A.A. performed writing review and editing. All authors have read and agreed to the published version of the manuscript.

## Data Availability

The data that support the findings of this study are available from the corresponding author upon reasonable request.
